# Incidental Findings on Computerized Tomography Images of Trauma Cases

**DOI:** 10.34172/aim.2022.98

**Published:** 2022-09-01

**Authors:** Ahsen Kaya, Ender Senol, Cenk Eraslan, Ali Mert Karaca, Elif Durdagi

**Affiliations:** ^1^Ege University Faculty of Medicine, Department of Forensic Medicine, Izmir, Turkey; ^2^Ege University Faculty of Medicine, Department of Radiology, Izmir, Turkey

**Keywords:** Computed tomography, Incidental finding, Medicolegal, Trauma

## Abstract

**Background:**

This study aimed to evaluate the characteristics of incidental findings (IFs) on computed tomography (CT) scans of trauma admissions, examine associations between IFs and gender and age-groups, and discuss the management strategies.

**Methods:**

The CT reports were retrospectively reviewed to evaluate IFs. Cases were divided into five age-groups (0–19, 20– 39, 40–59, 60–79,≥80). IFs were classified as *"Group 1"*: congenital anomalies that do not require further investigation, non-degenerative/minor degenerative findings; *"Group 2"*: findings that do not require immediate intervention, require outpatient follow-up/in case of symptoms; and *"Group 3"*: findings that require immediate intervention/further investigation.

**Results:**

There were 2385 CT scans and 1802 incidental findings (IFs) in 783 trauma cases. CT scans with IFs constituted 50.2%. The percentage of IFs was 75.6% in males and 24.4% in females, and they occurred in 4.8%, 27.6%, 44.3%, 20.9%, and 2.4% of age groups 1 to 5, respectively. Group 1 had 34.6%, group 2 had 54.6%, and group 3 had 10.8% IFs. There was not any significant association between the classification and gender or age-groups. In terms of organs, IFs of the thyroid and gall bladder & bile ducts were significantly higher among females (*P*=0.044 and *P*<0.001, respectively), while IFs in the head & neck region were significantly higher in males (*P*<0.001). Incidental findings in the kidney, liver, adrenal gland, and vascular structures differed significantly across age-groups (*P*<0.05).

**Conclusion:**

There were no significant relationships between the classification of IFs and gender or age-groups. However, the distribution of IFs was significantly associated with gender and age-groups in terms of organs. Healthcare professionals should consider this relationship when following up and treating patients.

## Introduction

 Traumas can cause minor injuries or result in loss of organs and limbs or even death. Rapid and accurate determination of trauma-related symptoms helps with correct diagnosis and treatment, and also forensic processes.

 Radiological imaging methods, which have become more specific with the developing technology, have a significant role in revealing the findings or diagnoses related to trauma. When the injuries related to trauma are defined by radiological methods, sometimes additional non-trauma-associated findings are also observed. In the literature, the frequency of incidental findings (IFs) varies between 30.6% and 53.0%.^1-3^ Determination of incidental pathologies may be beneficial for the patient, such as for early diagnosis of cancer. Besides, redundant examinations for IFs that will not change the patient’s health status can cause unnecessary anxiety.^1,2^

 This study aimed to evaluate the characteristics of IFs on computed tomography (CT) scans of trauma admissions, examine the relationships of IFs with gender and age groups, and discuss the management strategies.

## Materials and Methods

###  Participants

 The medical documents of 3348 trauma cases, who were admitted to Ege University, Faculty of Medicine, Department of Forensic Medicine during the two-year period from January 1, 2017 to December 31, 2018, were retrospectively reviewed. For 1611 of them, CT scans were performed and reported by the Department of Radiology. Of these, 783 (48.6%) cases with IFs were included.

 Cases who were followed up and treated in another center after traumatic injuries, those who did not undergo CT scan, and those without CT scan reports were excluded.

###  Data Management and Analysis

 Online access to the CT reports was provided from the hospital information system. Gender, age at the time of the event, type of CT scans, type of injury, injured body regions, and IFs were evaluated. The cases were divided into five age groups (0–19, 20–39, 40–59, 60–79, 80 and over). Age was included in all analyses according to this categorization, but the numerical age was summarized only in the text with mean ± standard deviation and range as descriptive statistics.

 All data were reported as frequencies and percentages with 95% confidence interval (CI) calculated using Wilson’s method.^4^ IBM SPSS Statistics 25.0 (IBM SPSS Statistics for Windows, Version 25.0. Armonk, NY: IBM Corp.) and R (R software, version 4.0.5, package: arsenal, R Foundation for Statistical Computing, Vienna, Austria; https://www.r-project.org/) were used for statistical analyses in this study. For all analyses, a *P* value less than 0.05 was considered statistically significant.

 The Chi-square test (or its Monte Carlo *P* values) was used to assess the association between two categorical variables.^5^ The adjusted residuals were used to determine which cells were responsible for a significant Chi-square result on r × c tables.^6^ They are distributed approximately normally under the independence null hypothesis of the Chi-square test, so they were represented in the text with z-scores. Also, when a positive z-score is more than 1.96, it means that the observed frequency in that cell is more than expected. Additionally, the linear-by-linear association test was used when analyzing crosstabs of two ordinal variables (or ordinal vs. binary variable).

###  Classification of Incidental Findings

 There are various classifications in the literature about IFs in imaging diagnostics tests.^1-3,7-15^ In a study conducted in Iran, IFs on brain CT were divided into two groups: intracranial findings with and without clinical symptoms.^7^ In another study, IFs on whole-body CTs were classified in two groups as findings that require urgent intervention or further examination and findings that do not require further investigation.^8^ In a pediatric age group study, IFs detected on brain CT were classified into three groups: requiring immediate evaluation and treatment, requiring outpatient follow-up, and not requiring specific follow-up or intervention.^9^

 In this study, considering the classifications used in the literature,^7-9^ IFs were classified into three groups:

- Group 1: Findings that do not require further investigation, congenital anomalies, and non-degenerative or minor degenerative findings.
- Group 2: Findings that do not require immediate intervention, require outpatient follow-up, or require follow-up or treatment in case of symptoms.
- Group 3: Findings that require immediate intervention or further investigation.

## Results

 Of the 783 cases included in this study, 606 (77.4%) were male and 177 (22.6%) were female. The mean age was 41.63 ± 16.35 years (range 1–93 years).

 The types of injuries were out-of-vehicle traffic accidents (n = 254, 32.4%; 95% CI, 29.3%-35.8%), in-vehicle traffic accidents (n = 212, 27.1%; 95% CI, 24.1%–30.3%), batteries (n = 170, 21.7%; 95% CI, 19.0%–24.7%), stab injuries (n = 43, 5.5%; 95% CI, 4.1%–7.3%), falls from heights (n = 33, 4.2%; 95% CI, 3.0%–5.9%), occupational injuries (n = 32, 4.1%; 95% CI, 2.9%–5.7%), and firearm injuries (n = 29, 3.7%; 95% CI, 2.6%–5.3%).

 One or more (max. 5) injured body regions were observed in 783 patients. Also, there were a total of 1530 affected anatomical regions. Of these, the cranial region (n = 452, 29.5%; 95% CI, 27.3%–31.9%), lower extremities (n = 308, 20.1%; 95% CI, 18.2%–22.2%), chest (n = 283, 18.5%; 95% CI, 16.6%–20.5%), and upper extremities (n = 258, 16.9%; 95% CI, 15.1%–18.8%) were the most frequently injured regions. The associations between injured body region and the type of injury are shown in Table 1.

**Table 1:** Association between Injured Body Regions and Types of Injury

**Types of injury** ***P*** **<0.001** ^*^		**Cranial**	**Chest**	**Lower Extremities**	**Upper Extremities**	**Spinal** **Column & Cord**	**Abdomen**	**Unidentified**	**Total**
Out-of-vehicle** (n = 254, 32.4%)	n	130^a^	92	167^b^	95	45	36	4	569
	%	28.8	32.5	54.2	36.8	41.7	35.6	20.0	37.2
In-vehicle (n = 212, 27.1%)	n	111	87^b^	56^a^	57	36^b^	18	12	377
	%	24.6	30.7	18.2	22.1	33.3	17.8	60.0	24.6
Batteries (n = 170, 21.7%)	n	156^b^	41^a^	23	55	7^a^	15	1	298
	%	34.5	14.5	7.5	21.3	6.5	14.8	5.0	19.5
Stab injuries (n = 43, 5.5%)	n	13^a^	21^b^	12	11	—	15^b^	—	72
	%	2.9	7.4	3.9	4.3	—	14.9	—	4.7
Falls from heights (n = 33, 4.2%)	n	16^a^	17	17	18	9	5	1	83
	%	3.5	6.0	5.5	7.0	8.3	5.0	5.0	5.4
Occupational injuries (n = 32, 4.1%)	n	17	12	11	10	9^b^	5	2	66
	%	3.7	4.2	3.6	3.8	8.3	5.0	10.0	4.3
Firearm injuries (n = 29, 3.7%)	n	4^a^	10	18^b^	8	1	6	—	47
	%	0.9	3.5	5.8	3.1	0.9	5.9	—	3.1
Others*** (n = 10, 1.3%)	n	5	3	4	4	1	1	—	18
	%	1.1	1.0	1.3	1.6	0.9	1.0	-	1.2
Total	n	452	283	308	258	108	101	20	1530
	%	100.0	100.0	100.0	100.0	100.0	100.0	100.0	100.0

^a^Negative significant z-score. ^b^Positive significant z-score. ^*^Monte Carlo (2-sided) *P* value. ^**^Pedestrians (n = 137), Motorcycle accidents (n = 117). ^***^Crush and incisive injury (n = 1), Unidentified (n = 9).

 There were 2385 CT scans for 783 trauma cases included in this study. Cranial CT scan was performed in 85.4% (n = 669) of the cases, cervical CT scan in 80.2% (n = 628), whole-body CT scan in 70.0% (n = 548), extremity CT scan in 23.0% (n = 174), maxillofacial CT scan in 6.8% (n = 161), lumbar vertebral CT scan in 2.6 (n = 62), thoracic vertebral CT scan in 2.4% (n = 58), abdominal CT scan in 2.1% (n = 50), and chest CT scan in 1.5% (n = 35). The number of CT scans with IFs was 1197 (50.2%) in this study, with 342 CT scans exhibiting > 1 IF (201 exhibiting 2, 74 exhibiting 3, 39 exhibiting 4, and 28 exhibiting ≥ 5 findings). The number of cases with IFs on these CT scans was 312.

 A total of 1802 IFs were observed in 783 cases. There was more than one IF in 453 cases. The percentage of IFs was 75.6% (n = 1363; 95% CI, 73.6%–77.6%) for males and 24.4% (n = 439; 95% CI, 22.4%–26.4%) for females, and it was 4.8% (n = 85; 95% CI, 3.8%–5.8%), 27.6% (n = 498; 95% CI, 25.6%–29.7%), 44.3% (n = 800; 95% CI, 42.1%–46.7%), 20.9% (n = 377; 95% CI, 19.1%–22.9%) and 2.4% (n = 42; 95% CI, 1.7%–3.1%) for each respective age group. The three body regions with the highest incidence of incidental pathologies were the abdominopelvic (n = 605, 33.6%; 95% CI, 31.4%–35.8%), head & neck (n = 426, 23.6%; 95% CI, 21.7%–25.7%), and spinal column & cord region (n = 352, 19.5%; 95% CI, 17.8%–21.4%), respectively. Furthermore, 5.4% (n = 98) of the IFs were congenital and 94.6% (n = 1704) were acquired.

 Incidental findings in the head and neck region were significantly higher in males (*P* < 0.001, χ^2^=12.343). There was no statistically significant difference in other regional IFs according to gender.

 In terms of the organs, the IFs detected in the thyroid, and gall bladder & bile ducts were significantly higher in females (*P* = 0.044, χ^2^ **=**4.043; *P* < 0.001, χ^2^ **=**12.456). The distribution of IFs detected in organs/systems by gender is shown in Table 2.

**Table 2:** Distribution of Incidental Findings Detected in Organs/Systems by Gender

**Organs/System**s	**Female**		**Male**		**Total**		χ^2^	***P*** ** Value**^a^
	**(n=177)**		**(n=606)**		**(n=783)**			
	**n**	** %**	**n**	**%**	**n**	**%**		
Lung	40	22.6	153	25.2	193	24.6	0.517	0.472
Kidney	34	19.2	138	22.8	172	22.0	1.015	0.314
Liver	40	22.6	100	16.5	140	17.9	3.468	0.063
Vascular system	24	13.6	75	12.4	99	12.6	0.174	0.677
Uterus/Ovary	40	22.6	-	-	40	5.1	144.322	**<0.001**
Adrenal Gland	6	3.4	33	5.4	39	5.0	1.223	0.269
Spleen	6	3.4	29	4.8	35	4.5	0.625	0.429
Thyroid	11	6.2	18	3.0	29	3.7	4.043	**0.044**
Gall bladder & bile ducts	14	7.9	14	2.3	28	3.6	12.456	**<0.001**
Lymphatic system	7	4.0	19	3.1	26	3.3	0.287	0.592
Lower gastrointestinal tract	7	4.0	19	3.1	26	3.3	0.287	0.592
Prostate	-	-	23	3.8	23	2.9	6.921	**0.009**
Heart	8	4.5	13	2.1	21	2.7	2.959	0.085
Bladder and lower urinary tract	1	0.6	10	1.7	11	1.4	1.165	0.280
Pancreas	5	2.8	3	0.5	8	1.0	-	0.017*
Trachea	-	-	5	0.8	5	0.6	1.470	0.225
Upper gastrointestinal tract	2	1.1	2	0.3	4	0.5	1.725	0.189
Thymus	-	-	2	0.3	2	0.3	0.586	0.444
Testicles	-	-	2	0.3	2	0.3	0.586	0.444
Breast	1	0.6	-	-	1	0.1	1.470	0.225

^a^Monte Carlo (2-sided) *P* value. ^*^Fisher Exact test (2-sided).

 The comparison of IFs detected in organs/systems according to age groups is shown in Table 3. The rate of IFs in the liver, kidney, adrenal gland, and vascular structures increased significantly with age (*P* = 0.011, *P* < 0.001, *P* = 0.004, *P* < 0.001, respectively).

**Table 3:** Distribution of Incidental Findings Detected in Organs/Systems According to Age Groups

**Organs/Systems**	**0–19**		**20–39**		**40–59**		**60–79**		**≥80**		**Total**		***P*** ** Value**^a^
	**(n=56)**		**(n=304)**		**(n=307)**		**(n=105)**		**(n=11)**		**(n=783)**		
	**n**	**%**	**n**	**%**	**n**	**%**	**n**	**%**	**n**	**%**	**n**	**%**	
Lung	9	16.1	62	20.4	89	28.9	29	27.6	4	36.4	193	24.6	**0.007**
Kidney	2	3.6	44	14.5	87	28.3	35	33.3	4	36.4	172	21.9	**<0.001**
Liver	6	10.7	41	13.5	68	22.1	25	23.8	-	-	140	17.9	**0.011**
Vascular system	2	3.6	19	6.3	40	13.0	32	30.5	6	54.5	99	12.6	**<0.001**
Uterus/Ovary	7	12.5	19	6.3	10	3.3	4	3.8	-	-	40	5.1	**0.009**
Adrenal gland	1	1.8	6	1.9	24	7.8	7	6.7	1	9.1	39	4.9	**0.004**
Spleen	3	5.4	15	4.9	11	3.6	5	4.7	1	9.1	35	4.5	0.827
Thyroid	-	-	6	1.9	17	5.5	5	4.7	1	9.1	29	3.7	**0.012**
Gall bladder & bile ducts	1	1.8	4	1.3	13	4.2	10	9.5	-	-	28	3.5	**0.001**
Lymphatic system	-	-	5	1.6	16	5.2	4	3.8	1	9.1	26	3.3	**0.018**
Lower gastrointestinal tract	-	-	8	2.6	13	4.2	4	3.8	1	9.1	26	3.3	0.082
Prostate	-	-	3	1.0	11	3.6	7	6.7	2	18.2	23	2.9	**<0.001**
Heart	-	-	2	0.7	6	1.9	12	11.4	1	9.1	21	2.7	**<0.001**
Bladder & lower urinary tract	-	-	2	0.7	5	1.6	3	2.9	1	9.1	11	1.4	**0.011**
Pancreas	-	-	-	-	2	0.7	6	5.7	-	-	8	1.0	**<0.001**
Trachea	2	3.6	-	-	1	0.3	2	1.9	-	-	5	0.6	**1.000**
Upper gastrointestinal tract	-	-	-	-	2	0.7	2	1.9	-	-	4	0.5	**0.075**
Thymus	-	-	2	0.7	-	-	-	-	-	-	2	0.2	-
Testicles	-	-	1	0.3	-	-	1	0.9	-	-	2	0.2	**-**
Breast	-	-	-	-	1	0.3	-	-	-	-	1	0.1	**-**

^a^Monte Carlo (2-sided) *P* value.

 The most common IF was degenerative changes in the vertebrae (n = 288, 36.8%; 95% CI, 33.5%–40.2%), followed by sinusitis (n = 203, 25.9%; 95% CI, 23.0%–29.1%), renal cyst (n = 131, 16.7%; 95% CI, 14.3%–19.5%) and lung nodule (n = 97, 12.4%; 95% CI, 10.3%–14.9%).

 According to the classification of IFs, there were 624 group 1 findings (34.6%; 95% CI, 32.5%–36.9%), 983 group 2 findings (54.6%; 95% CI, 52.2%–56.8%), and 195 group 3 findings (10.8%; 95% CI, 9.5%–12.3%). Incidental findings and their classifications are shown in Tables 4, 5, and 6.

**Table 4:** Incidental Findings in Group 1

**Group 1 (n=624)**			**n**	**%** ^*^
Head & Neck (n = 141)	Mucous retention cysts		73	9.3
	Age-related atrophic and ischemic changes in the brain		43	5.5
	Basal ganglia calcification		7	0.9
	Cavum septum pellucidum et vergae		4	0.5
	Intracranial lipoma		3	0.4
	Variational mega cisterna magna		3	0.4
	Eagle Syndrome		3	0.4
	Arachnoid granulations		1	0.1
	Hyperostosis frontoparietalis interna		1	0.1
	Platybasia		1	0.1
	Pyramidal lobe of the thyroid		1	0.1
	Cervical paravertebral intramuscular lipoma		1	0.1
Thorax (n = 84)	Atelectatic changes in the lungs		75	9.6
	Accessory lobe of the lung		2	0.3
	Azygos lobe		2	0.3
	Retained thymic tissue		2	0.3
	Accessory fissure of the lung		1	0.1
	Accessory papillary muscle		1	0.1
	Hypoplastic costa		1	0.1
Abdominopelvic (n = 47)	Accessory spleen		24	3.1
	Pancreatic atrophy		5	0.6
	Horseshoe kidney		3	0.4
	Phrygian cap		3	0.4
	Liver atrophy		2	0.3
	Adrenal myelolipoma		2	0.3
	Patent urachus		2	0.3
	Renal agenesis		1	0.1
	Variational enlargement of the renal pelvis		1	0.1
	Extrarenal pelvis		1	0.1
	Pancreatic lipoma		1	0.1
	Hepatic vascular malformation		1	0.1
	Uterine duplication anomaly		1	0.1
Vertebrae (n = 325)	Degenerative changes at vertebral bodies		288	36.8
	Vertebral fusion anomalies		26	3.4
	Spondylolisthesis		6	0.8
	Paravertebral ligament calcification		2	0.3
	Bone cyst		2	0.3
	Butterfly vertebra		1	0.1
Extremities (n = 13)	Upper extremity (n = 1)	Bone cyst	1	0.1
	Lower extremity (n = 12)	Bone cyst	8	1.0
		Epin calcanei	3	0.4
		Intraosseous lipoma of the ilium	1	0.1
Vascular structures (n = 14)	Vein (n = 6)	A normal variant of the portal vein	3	0.4
		Nutcracker syndrome	2	0.3
		Retroaortic renal vein	1	0.1
	Artery (n = 8)	Accessory renal artery	2	0.3
		Basilar artery fenestration	1	0.1
		Aberrant subclavian artery	1	0.1
		Right-sided aortic arch	1	0.1
		A normal variant of the hepatic artery	1	0.1
		Wilkie Syndrome	1	0.1
		The anatomical variant of the posterior tibial artery	1	0.1

^*^The percentages were calculated over the total number of cases (n = 783).

**Table 5:** Incidental Findings in Group 2

**Group 2 (n=983)**			**n**	**%** ^*^
Head & Neck (n = 252)	Sinusitis		203	25.9
	Osteoma/osteotome		11	1.4
	Arachnoid cyst		10	1.3
	Thyroid nodule ( < 1 cm)		7	0.9
	Tracheal diverticulum		5	0.6
	Fibrous dysplasia		4	0.5
	Otomastoiditis		3	0.4
	Periapical cyst		2	0.3
	Ventricular dilation		1	0.1
	Calcification of the falx cerebri		1	0.1
	Otitis media		1	0.1
	Siyalolitiazis		1	0.1
	Benign lesion located in the frontal bone		1	0.1
	Hemangioma in the sphenoid bone		1	0.1
	Fibroma of the parietal bone		1	0.1
Thorax (n = 142)	Pulmonary nodule		97	12.4
	Cardiomegaly		15	2.0
	Fibrotic changes/calcified granuloma at the lungs		14	1.8
	Bronchiectasis		8	1.0
	Left ventricular hyperplasia		2	0.3
	Sarcoidosis		2	0.3
	Hydatid cyst at the lung		1	0.1
	Calcification of the valves		1	0.1
	Benign rib lesion		1	0.1
	Esophageal diverticulum		1	0.1
Abdominopelvic (n = 467)	Renal cyst		131	16.7
	Hepatosteatosis		61	7.8
	Nephrolithiasis		40	5.1
	Ovarian cyst		27	3.5
	Adrenal adenoma/nodule ( < 3 cm)		23	3.0
	Hepatic cysts		18	2.3
	Prostatic hyperplasia		18	2.3
	Hepatomegaly		17	2.2
	Gallstone		17	2.2
	Colonic diverticulum		16	2.1
	Hepatic hemangioma		16	2.1
	Inguinal hernia		15	2.0
	Calcified granuloma at the liver		13	1.7
	Myoma uteri		9	1.1
	Prostatic calcifications		7	0.9
	Hiatal hernia		5	0.6
	Renal atrophy		4	0.5
	Splenomegaly		3	0.4
	Splenic cysts		3	0.4
	Bladder diverticulum		3	0.4
	Splenic granuloma		2	0.3
	Renal angiomyolipoma		2	0.3
	Umbilical hernia		2	0.3
	Calyceal diverticula		1	0.1
	Hydatid cyst at the liver		1	0.1
	Liver cirrhosis		1	0.1
	Hepatic angiomyolipoma		1	0.1
	Polycystic ovary syndrome		1	0.1
	Corpus hemorrhagicum		1	0.1
	Ovarian teratoma		1	0.1
	Bochdalek hernia		1	0.1
	Duodenal diverticulum		1	0.1
	Enteric duplication cyst		1	0.1
	Hydrocele		1	0.1
	Varicocele		1	0.1
	Bladder stone		1	0.1
	Acalculous cholecystitis		1	0.1
	Pancreatic duct dilation		1	0.1
Vertebrae (n = 26)	Hemangioma		10	1.3
	Spondyloarthritis/sacroiliitis		8	1.0
	Scoliosis		6	0.8
	Sclerotic lesions		2	0.3
Extremities (n = 18)	Upper extremity (n = 2)	Osteochondroma	2	0.3
	Lower extremity (n = 16)	Sclerotic lesions	7	0.9
		Benign bone lesions	4	0.5
		Enchondroma	2	0.3
		Osteochondroma	2	0.3
		Liposclerosing myxofibrous lesion at the femur	1	0.1
Vascular structures (n = 78)	Calcification/atheroma plaques at the aorta		53	6.8
	Calcification/atheroma plaques at the coronary artery		8	1.0
	Median arcuate ligament syndrome		8	1.0
	Calcification/stenosis at the internal carotid artery		2	0.3
	Pelvic congestion syndrome		2	0.3
	Ductus diverticulum		1	0.1
	Calcification/atheroma plaques at the mesenteric artery		1	0.1
	Subclavian artery stenosis		1	0.1
	Celiac trunk stenosis		1	0.1
	Chronic dissection of the celiac trunk		1	0.1

^*^The percentages were calculated over the total number of cases (n = 783).

**Table 6:** Incidental Findings in Group 3

**Group 3 (n=195)**		**n**	**%** ^*^
Head & Neck (n = 33)	Hypodense lesion at the thyroid	10	1.3
	Thyroid nodule ( > 1 cm)	5	0.7
	Macrolobular heterogeneous appearance at the thyroid	4	0.5
	Intracranial lesion	3	0.4
	Meningioma	3	0.4
	Increase in thyroid size	2	0.3
	Thyroiditis	1	0.1
	Hydrocephalus	1	0.1
	Ventricular softening (Intracranial HT?)	1	0.1
	Hyperdense lesion at the sinus (fungal ball? tooth root?)	1	0.1
	Lytic/expansive lesion at the mandible	1	0.1
	Increase in inner-outer table distance of calvarial bones	1	0.1
Thorax (n = 45)	Mediastinal lymph node (short axis > 1 cm)	18	2.3
	Mosaic pattern of the lung	15	1.9
	Lung mass ( > 3 cm) / Malignancy suspicious lesion	6	0.8
	Pleural calcification	2	0.3
	Heart failure	1	0.1
	Thrombus at the left atrial auricula	1	0.1
	Esophageal dilatation	1	0.1
	Calcified nodule at the breast	1	0.1
Abdominopelvic (n = 91)	Hypodense lesion at the liver	22	2.9
	Adrenal gland hyperplasia	11	1.4
	Hypodense lesion at the kidney	8	1.0
	Abdominal lymph node (short axis > 1 cm)	6	0.8
	Increase in the bladder wall thickness	5	0.7
	Dilated bile ducts	5	0.7
	Hypodense lesion at the spleen	4	0.5
	Mesenteric panniculitis	4	0.5
	Intrahepatic arteriovenous shunt	3	0.4
	Increase in stomach wall thickness	3	0.4
	Adrenal gland mass ( > 3 cm)	2	0.3
	Increase at gallbladder diameter/wall thickness	2	0.3
	Thickening of the appendix wall	2	0.3
	Over mass ( > 5 cm)	2	0.3
	Hyperdense lesion at the liver	1	0.1
	Lesion at adrenal gland favoring metastasis	1	0.1
	Solid renal mass	1	0.1
	Metachronous/transitional cell tumor at the kidney	1	0.1
	Increase at column wall thickness	1	0.1
	Colon polyp	1	0.1
	Increase at rectal wall thickness	1	0.1
	Cystic lesion at the retrorectal space (lymphangioma?)	1	0.1
	Increase at terminal ileum wall thickness	1	0.1
	Contrast mucosal enhancement pattern of ileum and cecum	1	0.1
	Thickening of the endometrium	1	0.1
	Neoplasia suspicious lesion at the pancreas	1	0.1
	Metastasic lesion at sacrum and ilium	1	0.1
Vertebrae (n = 1)	Paget's disease	1	0.1
Vascular structures (n = 25)	Increased pulmonary trunk diameter	8	1.0
	Aortic diameter increase/aneurysmatic dilatation	7	0.9
	Tortuous appearance of the aorta	4	0.5
	Aneurysmatic dilatation at the renal artery	2	0.3
	Total occlusion of the hepatic artery	2	0.3
	Dissection at SMA	1	0.1
	Dilatation of the iliac artery	1	0.1

^*^The percentages were calculated over the total number of cases (n = 783).

 The association of classification of IFs with gender, age groups, and body regions is shown in Table 7. The only significant association was between the classification of IFs and body regions (*P* < 0.001).

**Table 7:** Comparison of the Classification of Incidental Findings and Gender, Age Groups, and Body Regions

		**Group 1**		**Group 2**		**Group 3**		**Total**		***P*** ** Value**
		**(n=624)**		**(n=983)**		**(n=195)**		**(n=1802)**		
		**n**	**%**	**n**	**%**	**n**	**%**	**n**	**%**	
Gender	Male	467	74.8	747	76.0	149	76.4	1363	75.6	0.573^*^
	Female	157	25.2	236	24.0	46	23.6	439	24.4	
Age groups	0-19	27	4.3	55	5.6	3	1.5	85	4.8	0.430^**^
	20-39	176	28.2	284	28.9	38	19.5	498	27.6	
	40-59	258	41.4	443	45.1	99	50.8	800	44.3	
	60-79	145	23.3	185	18.8	47	24.1	377	20.9	
	≥ 80	18	2.8	16	1.6	8	4.1	42	2.4	
Regions	Head & Neck	141	22.6	**252** ^b^	**25.7**	**33** ^a^	**16.9**	426	23.6	< 0.001^**^
	Thorax	84	13.5	142	14.5	**45** ^b^	**23.1**	271	15.0	
	Abdominopelvic	**47** ^a^	**7.5**	**467** ^b^	**47.5**	**91** ^b^	**46.7**	605	33.6	
	Spinal column & cord	**325** ^b^	**52.1**	**26** ^a^	**2.6**	**1** ^a^	**0.5**	352	19.5	
	Extremities	13	2.1	18	1.8	**-** ^a^	**-**	31	1.7	
	Vascular structures	**14** ^a^	**2.2**	**78** ^b^	**7.9**	**25** ^b^	**12.8**	117	6.6	

^*^Linear by linear association test result. ^**^Monte Carlo (2-sided) *P* value. ^a^Negative significant z-score. ^b^Positive significant z-score.

## Discussion

 In this study, in which IFs were analyzed in trauma cases, 77.4% of 783 cases were male, and the mean age was 41.63 ± 16.35. In a study about the IFs in pediatric trauma patients, 68.5% of the cases were male.^14^ In a study by Sierink et al on IFs in trauma cases older than 18 years, males constituted 71.7%.^1^ In a similar study involving all age groups, the rate of males was 64.5%.^2^ Since males are more involved in work and social life than females, and males are more likely to engage in risky behaviors, the risk of being exposed to trauma is higher in males. On the other hand, some studies^3,15^ have reported that IFs are more common in females, andin some studies,^7,8,10,11^ there was no statistically significant relationship between IFs and gender.These differences across studies may have resulted from variations in the studied groups.

 In this study, the most common cause of trauma was traffic accidents, and out-of-vehicle traffic accidents were more common than in-vehicle traffic accidents. The widespread use of motor vehicles and non-compliance with traffic rules in Turkey may be a reason for this result. Statistically, thorax injuries in stab injuries and both thorax and spinal column & cord injuries in-vehicle traffic accidents were higher than expected. However, thorax injuries in batteries and spinal column & cord injuries in batteries and stab injuries were lower than expected. Similarly, lower extremity injuries were higher than expected in firearm injuries and out-of-vehicle traffic accidents, whereas they were lower than expected in batteries and in-vehicle traffic accidents. Also, cranial injuries were higher than expected in batteries, and lower than expected in falls from heights, stab injuries, out-of-vehicle traffic accidents and firearm injuries. Lastly, abdominal injuries were higher than expected in stab injuries. These data show that the type of trauma greatly affects the injured regions.

 In the present study, the most frequently injured area was the cranial region, and the majority of the 2385 CTs were cranial (85.4%) and cervical vertebral CTs (80.2%). The most frequently injured area^16,17^ and the most common types of CTs were compatible with the literature.^3,18^ The reason for these should be that CT scans have advantages in rapidly detecting emergency interventions, especially for head and neck traumas. Besides, the high number of CTs may be due to defensive medical practices. Unfortunately, there has been an increase in medical malpractice claims in Turkey.

 Approximately ¼ of cases (n = 453) had more than one IF consistent with the literature.^1,3,10-13,18^ Besides, as per the literature, IFs in the abdominopelvic region were the most common findings in trauma cases.^1,3,10,11,18^ Due to the presence of many organs in this region, the number of IFs detected in this region should be high.

 In the present study, the most common IFs were degenerative changes in the vertebrae, sinusitis, kidney cyst, and lung nodule, respectively. In the literature, the most common IFs were renal cysts,^1,3,12,18^ degenerative changes in the spine,^2,13^ and calcifications in the brain.^11^ Although the results of this study are generally compatible with the literature, some studies did not include IFs such as degenerative joint diseases, sinusitis, and age-related cerebral atrophy; in other words, the differences in the inclusion and exclusion criteria across studies might have led to differences in their findings.

 Incidental findings in the head and neck regions were significantly higher in males. Also, IFs of the thyroid, gallbladder, and bile ducts were significantly higher in females. There have been studies showing a gender relationship with tumors and diseases of the mentioned regions and organs. Since male smokers continue to outnumber female smokers and oral HPV infection is more frequent in males, head and neck cancer is more common among males.^19,20^ On the other hand, gall bladder and biliary diseases and thyroid problems have been 5 to 8 times more common in females, with pregnancy, estrogen, hormone replacement therapies in postmenopausal women and oral contraceptives noted as risk factors.^21-23^ These results are in line with the literature. However, when the classification of IFs was compared by gender, no statistically significant difference was found (*P* = 0.573).

 In this current study, the IFs especially in the vascular system, kidney, adrenal gland, prostate, and heart increased significantly with age. A statistically significant difference was observed between age groups and the classification of IFs (*P* = 0.430). Incidental findings in group 2 gradually decreased with increasing age, but IFs in group 1 and 3 increased with aging. The 40–59 years age group had the highest rates for group 1, 2 and 3. In similar studies, the incidence of IFs increased with age.^2,7,11,18^ Also, Kroczek et al stated that the severity of IFs (findings requiring urgent treatment/further investigation) rose with increasing age.^10^

 In the literature, there are several classification or scoring systems for IFs due to their clinical significance.^1-3,7-15^ In this current study, IFs were classified into three groups, based on the literature. More than half of the IFs (54.6%) were in group 2, and approximately 1/3 (34.6%) were in group 1. The relationship between the classification of IFs and body regions of the pathologies was statistically significant (*P* < 0.001). Vertebral pathologies were mostly observed to be in group 1, while abdominopelvic pathologies mostly fell in groups 2 and 3. Knowing this relationship can be a guide for physicians in evaluating and managing the process of IFs. In the literature, most of the IFs on CT scans were findings that do not require emergency intervention.^2,3,8-11,13,18^ Since IFs in group 3 are signs of malignancy, metastatic disease, or vascular aneurysm, early detection of these findings will reduce mortality and morbidity. Providing appropriate information to patients about the detected IFs will help prevent confusion and unnecessary examinations that may occur in the diagnosis and treatment process.^3^

 Nevertheless, many IFs may not be considered during the evaluation of traumatic cases but may be essential for the patient’s health in general. In cases investigated for trauma, IFs are more likely to be overlooked due to a greater focus on traumatic injuries. Also, there may be medicolegal issues in verifying the verbal information given to trauma patients. For these reasons, it is essential to pay attention to proper documentation, to give adequate and appropriate information, and to follow up on the cases.

 This study has some limitations. The current study was performed in a single center, so the results may not be applicable to other centers. There is not any standard classification of IFs in the literature. For this reason, a classification was developed based on the literature. Some IFs might not have been reported by radiologists when the trauma findings were more vital in some cases. There were not any medical records to show that information was given to the patients about the IFs, or recommendation of treatment, follow-up, or further tests. Therefore, information rates or follow-up and treatment processes of the cases could not be evaluated. Besides, it is not known whether the cases were already followed up for IFs before the trauma.
